# The synergic antitumor effects of paclitaxel and temozolomide co-loaded in mPEG-PLGA nanoparticles on glioblastoma cells

**DOI:** 10.18632/oncotarget.7896

**Published:** 2016-03-03

**Authors:** Yuanyuan Xu, Ming Shen, Yiming Li, Ying Sun, Yanwei Teng, Yi Wang, Yourong Duan

**Affiliations:** ^1^ State Key Laboratory of Oncogenes and Related Genes, Shanghai Cancer Institute, Renji Hospital, School of Medicine, Shanghai Jiao Tong University, Shanghai 200032, P. R. China; ^2^ Department of Ultrasound, Huashan Hospital, School of Medicine, Fudan University, Shanghai 200040, P. R. China

**Keywords:** nanoparticles, synergy, glioblastoma, paclitaxel, temozolomide

## Abstract

To get better chemotherapy efficacy, the optimal synergic effect of Paclitaxel (PTX) and Temozolomide (TMZ) on glioblastoma cells lines was investigated. A dual drug-loaded delivery system based on mPEG-PLGA nanoparticles (NPs) was developed to potentiate chemotherapy efficacy for glioblastoma. PTX/TMZ-NPs were prepared with double emulsification solvent evaporation method and exhibited a relatively uniform diameter of 206.3 ± 14.7 nm. The NPs showed sustained release character. Cytotoxicity assays showed the best synergistic effects were achieved when the weight ratios of PTX to TMZ were 1:5 and 1:100 on U87 and C6 cells, respectively. PTX/TMZ-NPs showed better inhibition effect to U87 and C6 cells than single drug NPs or free drugs mixture. PTX/TMZ-NPs (PTX: TMZ was 1:5(w/w)) significantly inhibited the tumor growth in the subcutaneous U87 mice model. These results indicate that coordinate administration of PTX and TMZ combined with NPs is an efficient method for glioblastoma.

## INTRODUCTION

Glioblastoma (GBM) is the most common and aggressive primary brain tumor [1, 2] with median survivals only 12-15 months [3, 4]. The treatment is still worldwide challenging. Given the post-operative radiotherapy could not prevent its recurrence and invasiveness towards surrounding brain tissue, the National Comprehensive Cancer Network (NCCN) 2012 suggested that patients with glioblastoma should consider chemotherapy except for with pilocytic astrocytoma or newly diagnosed ependymocytoma. The present treatment of GBM is multimodal involving surgery, radiotherapy and chemotherapy [5].

Temozolomide (TMZ) is the first agent in 20 years approved by the FDA to treat glioblastoma and has been one of the most commonly used anti-glioma agents with limited adverse effects [6, 7] due to its ability penetrating the blood brain barrier (BBB). The standard treatment is maximal surgical resection and maintenance treatment with temozolomide (TMZ), which could improve median and 5-year survival significantly [8]. However, the therapeutic effects of TMZ are far less enough. The O6-methylguanine-DNA methyltransferase (MGMT), a DNA repair protein involved in the resistance of tumor cells to alkylating agents, is also expressed in glioblastoma and contributes to the resistance to TMZ [9–11]. Thereby, combinational administrations of TMZ with other chemotherapeutics have been under study for improving the efficacy of glioblastoma therapies [12].

Paclitaxel (PTX) plays a crucial role in various tumors, as a kind of anti-microtubule drug. It has been the first-line therapy for patients with breast cancer and non-small cell lung carcinoma [13, 14]. PTX was reported a good apoptosis-inducing effect for glioblastoma cells *in vitro* [15, 16]. Moreover, the penetrating ability into the brain tumors of PTX was at least two orders of magnitude greater than carmustine and 5-fluorouracil, etc. [17].

Given TMZ belongs to cell cycle non-specific drugs, while PTX is a cell cycle specific drug which could restrain cell cycle at G2/M, we hypothesized that TMZ and PTX might exhibit synergistic effects if co-delivered simultaneously. One study indicated that PTX in combination with an alkylating agent could synergistically inhibit numerous types of cancers [18]. Particularly, PTX combined with cisplatin or TMZ has clear synergistic inhibitory effects against malignant glioblastoma cells *in vitro* [19]. But there were few reports on the optimal weight ratios of the two drugs co-delivered. Furthermore, the water solubility of PTX or TMZ is a serious limitation [20, 20]. Appropriate strategies were needed to co-deliver these two drugs effectively to brain. Among the multiple approaches, polymeric NPs seem to possess many advantages such as increased drugs reaching tumor sites, enhanced selectivity and the potential to co-deliver multiple agents simultaneously.

In this research, we prepared our nanoparticles with monomethoxy (polyethylene glycol) - poly (D, L-lactide-co-glycolide) (mPEG-PLGA). PLGA was approved for medical applications by FDA as a biocompatible and biodegradable polymer [21–23]. The mPEG could help the nanoparticles escape from reticuloendothelial system phagocytose and prolong its circulation in the bloodstream and further increase nanoparticles accumulation at the tumor tissues through the EPR effect [24–26]. We first determined the optimal weight ratio of PTX to TMZ for the composite nanoparticles delivery system. Then the dual drug-loaded mPEG-PLGA NPs was prepared with a double emulsion solvent evaporation method. The characteristics of the nanoparticles and their cytotoxicity profiles on U87 human malignant glioblastoma cells and C6 rat glioma cells were accessed. *In vivo* anti-tumor activity was evaluated with a BALB/c subcutaneous U87 glioblastoma xenograft model.

## RESULTS

### Characteristics of PTX/ TMZ-NPs

**Figure 1:**
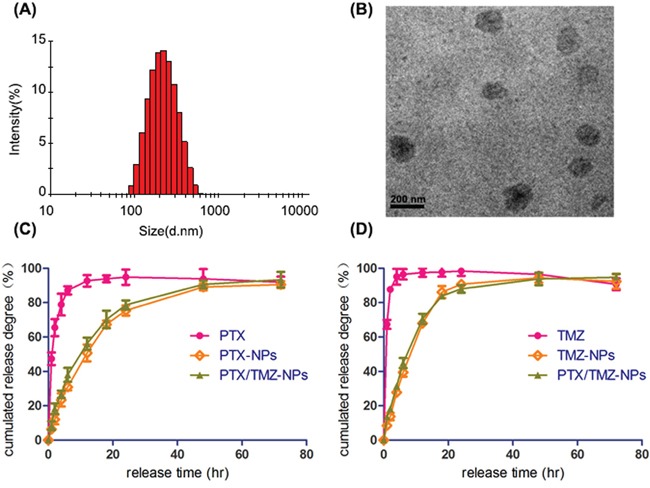
Characteristics of PTX/TMZ-NPs **A.** Particle size of PTX/TMZ-NPs. **B.** TEM image of PTX/TMZ-NPs. **C.** The PTX release profile. **D.** The TMZ release profile.

The mean size of PTX-NPs was 154.9 ± 21.3 nm prepared by the emulsion solvent evaporation method. TMZ-NPs and PTX/TMZ-NPs, prepared with the double emulsion solvent evaporation technique, achieved an average diameter of 172.9 ± 10.9 nm and 206.3 ± 14.7 nm (Figure 1A), respectively. The PTX/TMZ-NPs had a relatively smooth surface and uniform morphology (Figure 1B). The DL and EE of the NPs were listed in Table 1.

**Table 1:** The DL and EE of drug loaded NPs

NPs	DL %	EE %
PTX-NPs	0.917	92.5
TMZ-NPs	3.14	64.5
PTX/TMZ-NPs (PTX)	0.871	90.7
PTX/TMZ-NPs (TMZ)	3.15	65.2

The accumulated amount of PTX released from PTX solution achieved the maximum at 20 h, while from PTX-NPs or PTX/TMZ-NPs were both near 80 h (Figure 1C), showing apparent sustained characteristic and no significant difference between the two nanoparticles groups. Similarly, The TMZ released from the TMZ-NPs and PTX/TMZ-NPs showed remarkable sustained feature comparing with TMZ solution (Figure 1D). We could concluded that drugs-loaded nanoparticles exhibited a sustained release feature with decreasing amount of initial release, which might be explained by that drugs were gradually released with the dissolution of polymers.

### Synergic inhibition of PTX and TMZ on U87 cells and C6 cells

As expected, both PTX and TMZ demonstrated concentration-dependent inhibitory effects on U87 cells and C6 cells *in vitro* by MTT assay. The IC_50_ of PTX and TMZ for U87 cells at 48 h were 4.5 mg/L and 77.3 mg/L, respectively. C6 cell line was more sensitive to both the two drugs. The IC_50_ values of PTX and TMZ for C6 cells were 0.1 mg/L and 28.0 mg/L, respectively.

**Figure 2:**
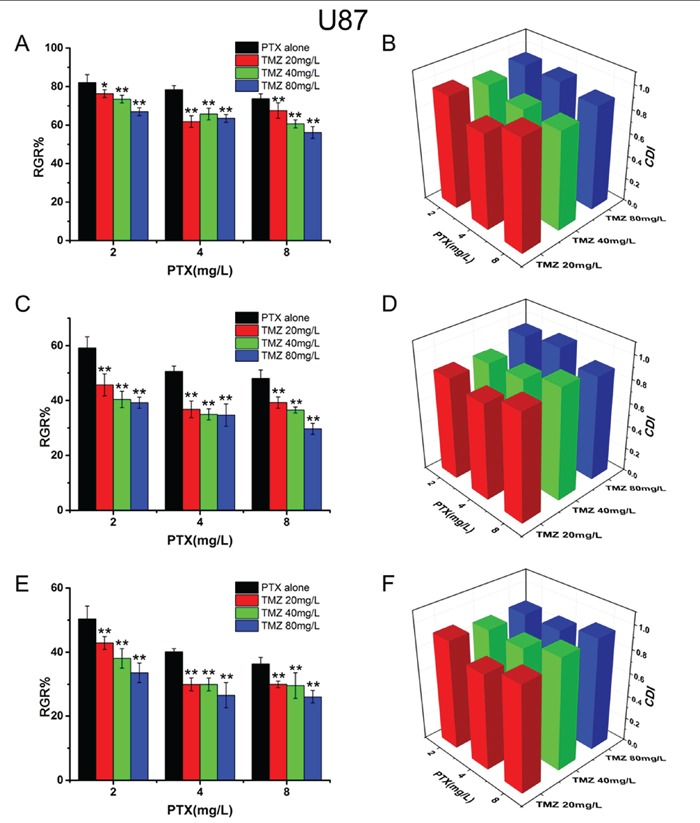
The synergistic inhibitory effects and CDI values of PTX and TMZ for U87 cells U87 cells treated with PTX and TMZ for 24 h **A.** 48 h **C.** 72 h **E.** CDI values of the combination groups for U87 cells for 24 h **B.** 48 h **D.** 72 h **F.** Data are presented as mean ± S.D. (error bar) of triplicate cultures. *P< 0.05, **P< 0.01, vs. PTX alone.

**Figure 3:**
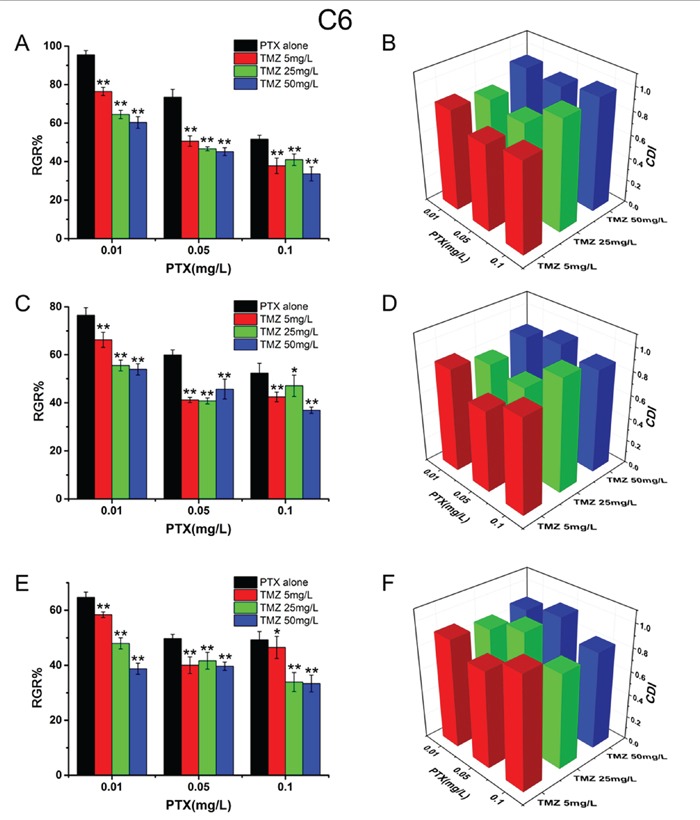
The synergistic inhibitory effects and CDI values of PTX and TMZ for C6 cells C6 cells treated with PTX and TMZ for 24 h **A.** 48 h **C.** 72 h **E.** CDI values of the combination groups for C6 cells for 24 h **B.** 48 h **D.** 72 h **F.** Data are presented as mean± S.D. (error bar) of triplicate cultures. *P< 0.05, **P< 0.01, vs. PTX alone.

To investigate the synergistic inhibitory effects of PTX and TMZ, both drugs at different concentrations were used to simultaneously treat cells based on the IC_50_ values. The results showed that TMZ increased the cytotoxicity of PTX on U87 (Figure 2) and C6 cells (Figure 3). CDI values were all less than 1, which meant PTX and TMZ had synergistic effects on both U87 cells and C6 cells.

The combination of PTX and TMZ on the concentrations of 4.0 mg/L and 20 mg/L, respectively showed a better synergic effect (lower CDI values) on U87 cells. We speculated that when the ratio of PTX to TMZ was 1:5(w/w) the synergic effect on U87 was the best. While the most remarkable synergic effect was observed on C6 cells when the concentrations were 0.05 mg/L and 5 mg/L, respectively. Therefore, the best synergic effect on C6 was obtained when the ratio of PTX to TMZ was 1:100(w/w). Nevertheless, increasing the concentrations of PTX or TMZ further did not yield statistically significant differences in CDI values.

### Combined inhibitory and apoptosis-inducing effects of PTX and TMZ co-loaded in mPEG-PLGA NPs on U87 cells and C6 cells

**Figure 4:**
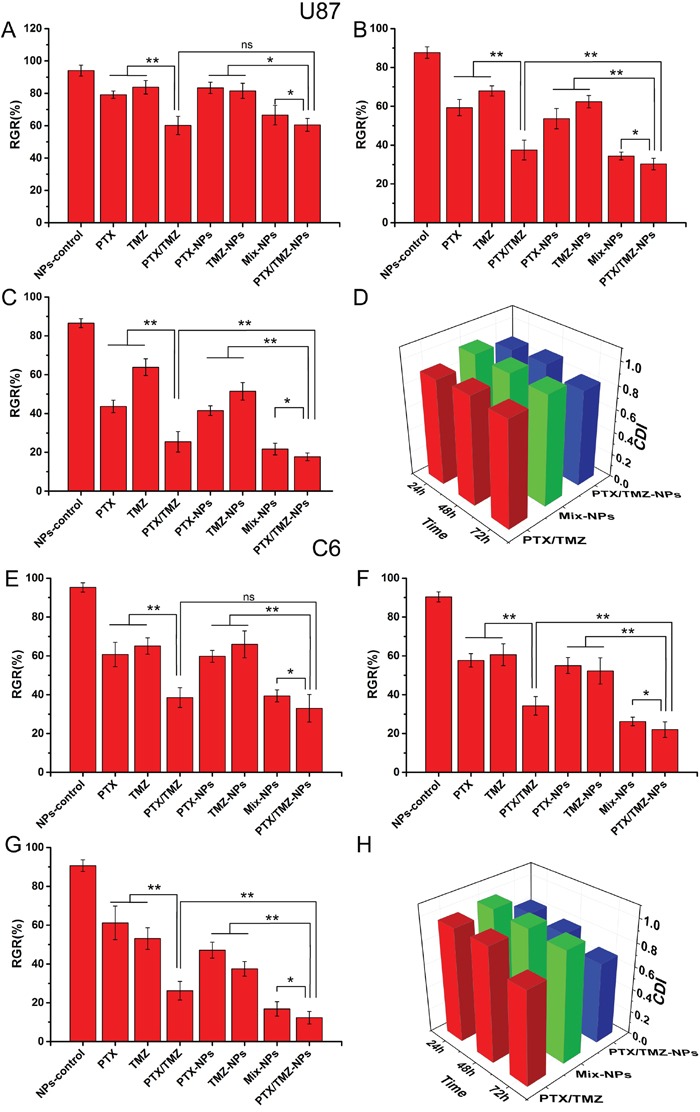
The synergistic inhibitory effects of PTX and TMZ co-loaded in mPEG-PLGA NPs on U87 cells and C6 cells U87 cells treated with PTX/TMZ-NPs and other comparative formulations for 24 h **A.** 48 h **B.** 72 h **C.** and CDI values of the combination groups **D.** C6 cells treated with PTX/TMZ-NPs and other comparative formulations for 24 h **E.** 48 h **F.** 72 h **G.** CDI values of the combination groups for C6 cells **H.** Data are presented as mean ± S.D. (error bar) of triplicate cultures. *P< 0.05, **P< 0.01, ns P>0.05.

Figure 4 indicated that the combined formulations, including the PTX/TMZ, Mix-NPs or PTX/TMZ-NPs, all presented synergic effects when incubated with U87 cells and C6 cells, respectively. PTX/TMZ-NPs exhibited a better synergic effect with statistically significant differences comparing with Mix-NPs. Nevertheless, PTX/TMZ-NPs showed significant differences to PTX/TMZ formulations only at 48 h and 72 h, but not at 24 h, which may be in accordance with the sustained release characteristic of NPs. The blank mPEG-PLGA NPs did not have any cytotoxic effects on the both cells.

**Figure 5:**
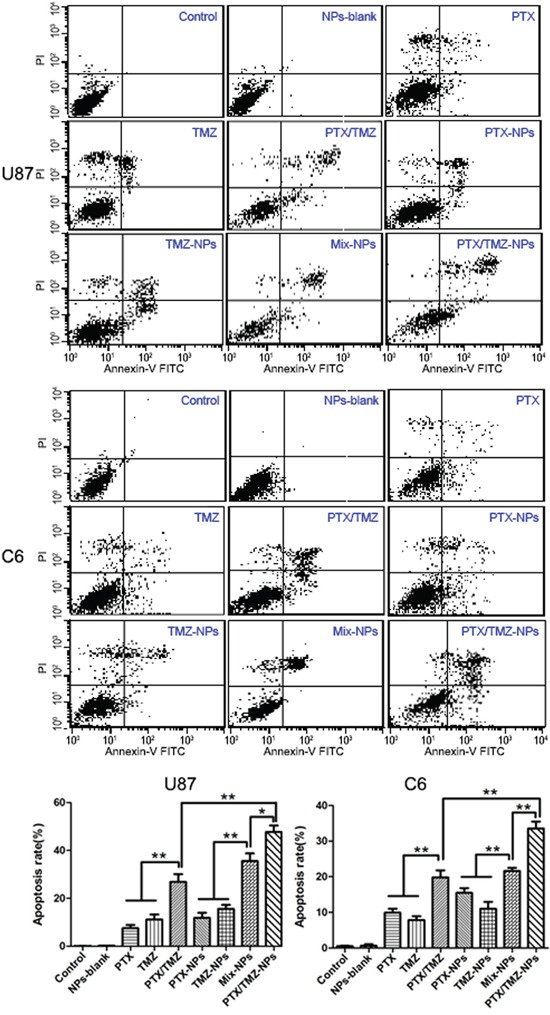
Flow cytometer analysis on cell apoptosis of U87 cells and C6 cells incubated 48 h with different formulations The PTX, PTX-NPs or PTX/TMZ-NPs solutions were at an equivalent concentration of 4 mg/L and 0.05 mg/L PTX for U87 cells and C6 cells, respectively; and the TMZ, TMZ-NPs or PTX/TMZ-NPs at an equivalent concentration of 20 mg/L and 5 mg/L TMZ respectively. Data are represented as mean ± S.D. (n=3). *P< 0.05, **P< 0.01.

Significantly different apoptotic rates were observed among various treatments at 48 h on U87 cells and C6 cells (Figure 5). Exactly as the proliferation inhibitory results demonstrated that statistically significant differences not only existed between single and dual drug groups, but also among the combined formulations groups, wherein the apoptosis rate of PTX/TMZ-NPs group was the highest.

### *In vivo* anti-tumor efficacy of PTX/TMZ-NPs

**Figure 6:**
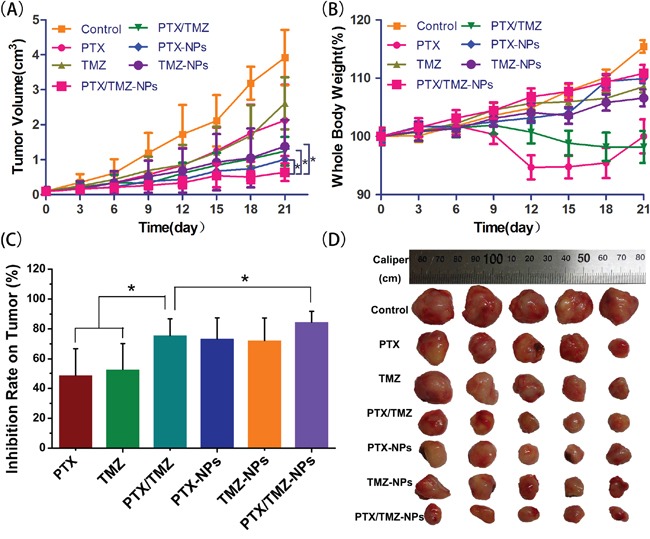
The anti-tumor efficacy of PTX/TMZ-NPs on nude mice bearing U87 xenograft glioblastoma at the experiment terminal (n=5) **A.** Tumor growth curves; mean ± SD. Significant differences found between the PTX/TMZ or PTX–NPs or TMZ-NPs and the PTX/TMZ-NPs groups, and labeled with *p < 0.05. **B.** Whole body weight; mean ± SD. **C.** Inhibition rate on tumors. **D.** Tumor xenografts alignment of each group taken out from the sacrificed mice at the study end point. *P< 0.05.

*In vivo* anti-tumor efficacy of PTX/TMZ-NPs was evaluated on the subcutaneous U87 model. As shown in Figure 6, the tumor sizes of therapeutic groups were all notably smaller than that of the control group (P<0.01). The inhibition effect of PTX/TMZ-NPs was the best. We also found that effects of dual drug groups were better than single drug groups, whatever in solution or in nanoparticles. The nanoparticles groups were better than PTX/TMZ solution but without significant difference. These results were in the same trend with the results *in vitro*.

However, the significant loss of whole body weight was observed in PTX or PTX/TMZ group indicating systemic toxicity (Figure 6B), which was coincident with other reports (23, 40). However, this did not happen in NPs groups. We hypothesized that the sustained release and tumor target of the NPs reduced the side-effects.

## DISCUSSION

Combination therapy is a common way of clinical oncology chemotherapy. Because the TMZ and PTX produce the antitumor effect by different routes, we were interested in their combined effect on tumor. In addition, the two drugs are of water soluble and fat soluble separately and their encapsulation in NPs can be achieved by a same method and should not affect each other. It was reported that glioma cells exhibit decreased glucose uptake and lactate production in response to treatment with TMZ; however, glucose metabolism was increased after Taxol treatment. And the glucose metabolism was decreased in the TMZ-resistant cells, but was increased in the Taxol-resistant cells (41). This might be a performance of synergy. The combination therapy of the two drugs were reported good effect in a phase I trial on melanoma (42).

The coefficient of drug interaction (CDI) was used to analyze the synergistically inhibitory effect of drug combinations. The CDI values less than, equal to or greater than 1 demonstrates that the drugs are synergistic, additive or antagonistic, respectively. Particularly, CDI values less than 0.7 indicate the agents are significantly synergistic [27–29]. A PTX thermo gel depot with TMZ and radiotherapy was reported on gliosarcoma *in vivo* [30]. But the CDI was not calculated. PTX and TMZ were reported to be co-loaded microsphere on C6 cells *in vitro* [31]. The optimal weight ratio was 1:1. While in our research the optimal ratio was 1:100 on C6 cells. The difference might be because of the different drug release rate in nanoparticles and microsphere.

Based on the synergic of the free drugs, we designed the co-loaded NPs. TMZ is water soluble while PTX is fat soluble. So we designed a hydrophobic shell with the PLGA block to capsulate PTX and the aqueous internal cavities to capsulate TMZ. The mPEG block provided hydrophilic outer shell for EPR effect. The two drugs were loaded in different positions of the NPs and their capsulation did not decrease than single-loaded.

The synergic relationship was observed not only between drug solutions, but also in co-loaded NPs, single-loaded and their mixture (P<0.05). At 24h, the superiority of NPs was not obvious. But after 48h, co-loaded NPs showed better inhibition effect than the mixed solution. This may be because of the sustained release of the NPs so that the drug concentration in cells rose more slowly in NPs groups than in the solution group. But this sustained release maintained drug concentration in cells for a longer time to get better inhibition effect. The co-loaded NPs showed better effect than single-loaded NPs mixture. We supposed this may be because the single-loaded NPs amount was larger than the co-loaded NPs at the same drug concentrations. The cell uptake and efflux is a reversible process in equilibrium. Thus the large amount tends to be transported more, so that the drugs in cells were not equal to the co-loaded NPs group. The detail reasons will be researched in our future work.

The mPEG block set up hydrophilic outer shell, which provided long-circulation character and enhanced NPs distribution in tumor site *in vivo* than the solution group [32, 33]. The sustained release characteristic of nanoparticles is essential not only for improving anti-cancer efficacy but also for reducing adverse-effects. PTX/TMZ-NPs would be a promising device for the long-term delivery of glioblastoma therapy.

## MATERIALS AND METHODS

### Materials

The 3-(4, 5-dimethylthiazol-2-yl)-2, 5-diphenyltetrazolium bromide (MTT) was purchased from Shanghai qcbio Science &Technologies co., Ltd.. Dimethyl sulfoxide (DMSO) and Pluronic F-68 (F-68) were purchased from Sigma-Aldrich (USA). Annexin V-FITC Apoptosis Detection Kit was obtained from Beyotime Institute of Biotechnology (Jiangsu, China). Monomethoxy (polyethylene glycol) - poly (D, L -lactide- co -glycolide) (mPEG-PLGA) (Mw=15140, mPEG Mw=5000, 20%, LA: GA 8:2) was synthesized in our laboratory [34]. Paclitaxel (PTX) and Temozolomide (TMZ) standard were from National Institutes for Food and Drug Control. PTX bulk drug was from Jiangsu YEW Biotechnology CO., Ltd (China). TMZ bulk drug was from Dalian Meilun Biotech Co., Ltd (China). Other reagents and solvents (AR grade) were purchased from Sinopharm Chemical Reagent Co., Ltd (China).

### Animals and cell lines

Female BALB/c nude mice (18 ± 2 g) were obtained from Shanghai SLRC Laboratory Animal Co., Ltd (China). C6 rat glioma cell line and the human malignant glioblastoma cell line U87 were purchased from Cell Bank of Chinese Academy of Sciences (Shanghai, China). All cell culture reagents were purchased from GIBIC Corporation (CA, USA).

### Nanoparticle preparations

The PTX-NPs were prepared using an emulsion solvent evaporation method [35, 36]. Briefly, 10 mg mPEG-PLGA was dissolved in 500 μL of PTX dichloromethane solution (PTX 0.2 mg/mL). The mixture was added into 5ml 0.5% F-68 solution and emulsified for 120 s (2s-2s-300w) by an ultrasonic processor (JY92-2D Ultrasonic cell crusher, Ningbo SCIENTZ biotechnical Co., Ltd). Then the emulsion was stirred at room temperature to remove the dichloromethane. The nanoparticles were then collected and freeze-dried for subsequent use.

A double emulsification solvent evaporation technique was used to prepare the TMZ-NPs [36, 37]. TMZ was dissolved in 80 uL of 0.1 M HCl (4.4 mg/mL) and added into 700 μL dichloromethane containing 7 mg mPEG-PLGA. The mixture was emulsified for 120s (2s-2s-300w) with an ultrasonic processor. Then the initial emulsion was poured into 5ml 1% F-68 solution quickly and emulsified again. The emulsion was then stirred at room temperature to remove the dichloromethane. The nanoparticles were then collected and freeze-dried for subsequent use.

The PTX/TMZ-NPs were prepared with the same double emulsion solvent evaporation method with the TMZ-NPs. TMZ solution (4.4 mg/mL) 80μL was added into 700 μL dichloromethane containing 7 mg mPEG-PLGA and PTX (0.1 mg/mL). Then the mixture was emulsified and poured into 5ml 1% F-68 solution and emulsified again. The dichloromethane was removed by stirred. The nanoparticles were then collected and freeze-dried for subsequent use.

### Characterization of nanoparticles

The size distribution and average diameters of PTX/TMZ-NPs were analyzed by Zetasizer IV analyzer (Malvern Zetasizer Nano ZS90, UK). The morphology was observed with Transmission electron microscopy (TEM) (H-800; Hitachi, Japan).

The concentration of PTX was determined by HPLC (Agilent 1200, USA) with a C_18_ chromatographic column (Zorbax SB-C_18_, 150×4.6 mm, 5 μm). The mobile phase was acetonitrile: 10 mmol/L NH_4_Ac solution (pH=5.0) 53:47 at a flow rate of 1.0 mL/min and the detection wavelength was 227 nm [38, 39]. The TMZ was monitored at a wavelength of 328 nm using an ultraviolet spectrophotometer (UV9000, AoYi Instrument Co., Ltd.) [20]. The free PTX was separated by centrifuge (5000r/min × 5min) and the precipitate was removed. The free TMZ was separated by ultracentrifugation (MWCO=10kDa) and the solution was collected as free TMZ. The encapsulation efficiency (EE) and Drug Loading rate (DL) were calculated as follows:

$$\text{EE}(\%)=(\frac{\text{weight of the drug in nanoparticles}}{\text{weight of the feeding drug}})\times 100\%$$

$$\text{DL}(\%)=\frac{\text{weight of the feeding drug}}{\text{weight of the nanoparticles}}\times 100\%$$

*In vitro* release study was investigated at 37°C in PBS buffer (pH 7.4, with 1mol/L sodium salicylate). Nanoparticles were suspended to 1 mL and dialyzed against 19 mL PBS using a dialysis tube (MWCO=3.5 kDa) with shaking at 80 rpm. At preset times, 200 μL of dialyzed solution were collected and the same volume of fresh buffer was added. PTX or TMZ concentrations were determined as described above.

### Synergic inhibition of PTX and TMZ on U87 cells and C6 cells

C6 cells and U87 cells were cultured in DMEM medium (GIBCO) supplemented with 10% (v/v) heat-inactivated fetal bovine serum (FBS) and 1% antibiotic solution (penicillin 100 U/mL and streptomycin 100 μg/mL) at 37°C in a humidified atmosphere of 95% air/5% CO_2_.

U87 cells and C6 cells were seeded in 96-well plates and cultured overnight. The medium was replaced with a series of concentrations of PTX (0.1, 0.25, 0.5, 1, 2.5, 5, 7.5, 10, 15, 25, 40, 50 mg/L) or TMZ (1, 2.5, 5, 10, 25, 50, 75, 150, 300, 600, 1200, 1500 mg/L) respectively. After further cultured for 24/48/72 hours, the medium was changed with fresh DMEM with MTT (0.5 mg/mL) for another 4 h. The resulting formazan was dissolved in 200 μL of DMSO and detected at 490 nm using a Microplate Reader (Bio-Rad Laboratories Inc, Hercules, CA, USA). The relative growth rate of the cells (RGR %) was determined by the following equation:

$$\text{RGR}(\%)=\frac{\text{OD}(\text{sample})-\text{OD}(\text{blank})}{\text{OD}(\text{control})-\text{OD}(\text{blank})}\times 100\%$$

IC_50_ values (the median inhibitory concentration) were obtained.

Based on the IC_50_ values, U87 cells and C6 cells were treated with PTX, TMZ, PTX plus TMZ (PTX/TMZ) or a control solution. CDI is calculated as follows: CDI=AB/ (A×B). The AB represents the RGR of the combination group. The A and B are the RGR of the single agent groups.

### Combined inhibitory and apoptosis-inducing effects of PTX and TMZ loaded in nanoparticles on U87 cells and c6 cells

U87 cells and C6 cells were plated in 12-well plates respectively and cultured overnight. The medium in the wells were refreshed and PTX or TMZ preparations were added in for 48 h. Then, the wells were divided into two parts. One part was changed with fresh DMEM with MTT and detected as above. The other wells were treated under instructions of apoptosis detection Kit (Annexin V-FITC, Byotime Co., Ltd.) and detected with a flow cytometry. The treatments were performed in triplicate, and the percentage of labeled cells undergoing apoptosis in each group was determined and calculated.

### *In vivo* antitumor activity

A U87 cell xenografts model was set up on nude mice by injecting suspension of U87 cells (5×10^6^ cells in 0.2 mL of saline) subcutaneously. When the tumors were about 90 ± 10 mm^3^, the mice (female, 18±2 g) were randomly divided into seven groups (n=5) and intravenously administered respectively with 200 μL of saline, PTX, TMZ, PTX/TMZ, PTX-NPs, TMZ-NPs or PTX/TMZ-NPs, at the dose of PTX 4 mg/kg [40, 43] and TMZ 20 mg/kg [44]. The treatments were repeated every two days. Tumor size was used to assess the efficacy of therapy. Two perpendicular diameters were measured with a caliper every three days until the mice were sacrificed. The tumor volume (V) was calculated as:

$$V({\text{mm}}^{3})=\frac{\text{length}\times {\left(\text{width}\right)}^{2}}{2}$$

Meanwhile, the whole body weight was simultaneously monitored as an evaluation of toxicity.

### Data analysis

Data were generated in multiples of triplicates for proper statistical analysis. Analysis of variance (ANOVA) was used within each treatment and applied among the groups. Results were expressed as mean ± standard deviation (SD). A probability (P) less than 0.05 was considered statistically significant.
